# Chk1 Inhibition Hinders the Restoration of H3.1K56 and H3.3K56 Acetylation and Reprograms Gene Transcription After DNA Damage Repair

**DOI:** 10.3389/fonc.2022.862592

**Published:** 2022-04-14

**Authors:** Nan Ding, Zhiang Shao, Fangyun Yuan, Pei Qu, Ping Li, Dong Lu, Jufang Wang, Qianzheng Zhu

**Affiliations:** ^1^ Key Laboratory of Space Radiobiology of Gansu Province and Key Laboratory of Heavy Ion Radiation Biology and Medicine of Chinese Academy of Sciences, Institute of Modern Physics, Chinese Academy of Sciences, Lanzhou, China; ^2^ College of Life Science, University of Chinese Academy of Sciences, Beijing, China; ^3^ Department of Radiology and Department of Molecular and Cellular Biochemistry, The Ohio State University, Columbus, OH, United States; ^4^ James Cancer Hospital and Solove Research Institute, The Ohio State University, Columbus, OH, United States; ^5^ Department of Oncology, The First Hospital of Lanzhou University, Lanzhou, China

**Keywords:** posttranslational modification (PMT), H3K56 acetylation, radiation, Chk1, chromatin assembly

## Abstract

H3K56 acetylation (H3K56Ac) was reported to play a critical role in chromatin assembly; thus, H3K56ac participates in the regulation of DNA replication, cell cycle progression, DNA repair, and transcriptional activation. To investigate the influence of DNA damage regulators on the acetylation of histone H3 and gene transcription, U2OS cells expressing SNAP-labeled H3.1 or SNAP-labeled H3.3 were treated with ATM, ATR, or a Chk1 inhibitor after ultraviolet (UV) radiation. The levels of H3.1K56ac, H3.3K56ac, and other H3 site-specific acetylation were checked at different time points until 24 h after UV radiation. The difference in gene transcription levels was also examined by mRNA sequencing. The results identified Chk1 as an important regulator of histone H3K56 acetylation in the restoration of both H3.1K56ac and H3.3K56ac. Moreover, compromising Chk1 activity *via* chemical inhibitors suppresses gene transcription after UV radiation. The study suggests a previously unknown role of Chk1 in regulating H3K56 and some other site-specific H3 acetylation and in reprograming gene transcription during DNA damage repair.

## Introduction

The maintenance of genomic integrity and stability is crucial for the growth, development, homeostasis, and survival of all organisms. However, genomic instability is continually induced by various exogenous and endogenous factors such as radiation, ecotoxic chemicals, and DNA replication. DNA repair pathways and the subsequent associated processes have evolved universally in all eukaryotic organisms to limit genomic instability (1). In general, the cellular DNA damage response (DDR) originates from DNA lesion recognition, followed by the initiation of a complex cellular signaling cascade to promote DNA repair, and these cascades include cell cycle arrest, which is aided by checkpoint activation. The DDR regulation is mainly organized by the following kinases in the phosphoinositide-3-kinase (PI3K)-related family of protein kinases (PIKKs): ataxia-telangiectasia mutated (ATM), ATM and RAD3-related (ATR), and DNA-dependent protein kinase, catalytic subunit (DNA-PKcs) (2). ATR-Chk1 and ATM-Chk2 are the two main signaling axes of the DDR network in mammals (3). For instance, the S317 and S345 residues of Chk1 are phosphorylated by the upstream kinase ATR when DNA damage is caused by radiation, replication stress or ecotoxic chemicals (4). Then, activated Chk1 will phosphorylate a variety of downstream substrates to modulate various cellular processes and the DNA damage response (DDR) (5).

In the DDR, posttranslational modifications (PTMs) of histones, such as acetylation, methylation, SUMOylation, and phosphorylation, play unique roles in controlling chromatin structure and gene activity (6, 7). Among these PTMs, the lysine acetylation of histones alters the accessibility of chromatin and offers a protein interaction platform during transcription, replication, and DNA repair (8, 9). Specifically, the acetylation of histone H3 at lysine 56 (H3K56ac) plays a critical role in regulating chromatin assembly during DNA synthesis and transcriptional activation, as this residue is located in the core of histone H3 at the nucleosome dyad (10, 11).

H3K56ac is a transient chromatin signal that turns over during transcription and DNA damage repair. In an unperturbed cell cycle, H3K56 of newly synthesized histones becomes acetylated during S-phase and disappears as cells progress through the G2 phase of the cell cycle (12). During the DDR, nucleosome destabilization and reassembly occur in response to genotoxic stress-induced DSBs (13). Because H3K56ac signals for the recovery of chromatin structure over repaired DNA, the presence of H3K56ac during DDR is a critical indicator that the DNA damage checkpoint is turning off, which allows cell cycle re-entry after DNA repair (9, 12). Tjeertes’ research and our previous study identified that H3K56ac and H3K9ac are rapidly diminished and subsequently restored in U2OS cells and HeLa cells after ionizing radiation (IR) and ultraviolet radiation (UVR) (14–16). Furthermore, cells lacking H3K56ac may be sensitive to genotoxic agent due to defects in chromatin assembly (17). However, the cellular regulation of H3K56 acetylation in relation to DDR activation and termination is not fully understood.

In mammals, the key histone H3–H4 chaperone anti-silencing factor 1 (Asf1) is essential for the acetylation of H3K56. Asf1 functions in both DNA replication-dependent and replication-independent nucleosome assembly (18). In higher eukaryotes, the protein kinases in the Tousled-like family (TLKs) are the upstream regulators of Asf1 homolog (19). TLK signaling promotes cellular histone supply in S phase by targeting histone-free Asf1 and stimulating its ability to shuttle histones to sites of chromatin assembly (20, 21). Importantly, TLKs are rapidly inactivated in response to genotoxic stress through phosphorylation by the checkpoint kinase Chk1 (22, 23). However, the regulatory effect of Chk1 on the acetylation of H3K56 during the DDR remains unclear.

Mammalian histone H3 has several variants, namely, H3.1, H3.2, H3.3, H3t/H3.4, H3.5, H3.6, H3.8, H3.Y, H3.X, and CENP-A. The replicative variant H3.1 and the replacement variant H3.3 are arguably some of the better-studied histone variants (24, 25). The accumulation of both new H3.1 and H3.3 variants at UV-damaged DNA regions was detected in U2OS cells (26). However, the H3K56 acetylation status, levels, and functionality of individual histone variants H3.1 and H3.3 remain to be explored. Whether and how DDR regulators may be linked with the recovery of H3.1K56ac and H3.3K56ac to DDR termination and aftermath transcription recovery and cell cycle progression are critical and open questions.

In this study, we investigated the possible influence of the DDR regulators ATM, ATR and Chk1 on the acetylation of H3K56, H3K27, H3K14, and H3K9 after UVR. Our data revealed a previously unknown regulatory effect of Chk1 on H3K56 acetylation in response to UVR-induced DNA damage. Using chemical inhibition and mRNA sequencing as tools, we further explored transcriptional reprogramming during UVR-induced DDR and revealed a regulatory role of Chk1 in cellular transcription linked to H3K56 acetylation.

## Materials and Methods

### Cell Lines, Chemicals, and Antibodies

The stable U2OS cell lines expressing H3.1-SNAP or H3.3-SNAP were generated and previously described by Dunleavy et al. (27). Of note, SNAP tag is a 20 kilo-Dalton (kDa) mutant of the DNA repair protein O6-alkylguanine-DNA alkyltransferase, and the SNAP-tagged histone variants enable visualization and detection of histones and their modifications in cooperation in chromatin, for example, the H3.3 and H3K4Me2 at centromere.

Human U2OS cells (ATCC) were cultured in Dulbecco’s modified Eagle medium (DMEM) (Thermo Fisher Scientific, Waltham, MA 02451, USA) supplemented with 10% fetal bovine serum (FBS) (Thermo Fisher Scientific) and 1% penicillin and streptomycin at 37°C in a humidified atmosphere of 5% CO_2_. The H3.1- and H3.3-SNAP U2OS cell lines were also cultured in DMEM under the same conditions. For the serum starvation experiment, cells were seeded and grown to 70% confluence and then the medium was changed to serum-free DMEM medium for 24 h.

The ATM inhibitor KU55933, the Chk1 inhibitor UCN-01, and the Chk1&Chk2 inhibitor AZD7762 were obtained from Sigma-Aldrich (St. Louis, MO 63103, USA), while the ATR inhibitor VE-821, the Chk1 inhibitor MK8776, and the ATM & ATR inhibitor AZD6738 were obtained from Cayman Chemical company (Ann Arbor, Michigan 48108, USA). The Chk1 inhibitor Ly2606368 was obtained from Selleck Chemicals (Houston, TX 77014, USA).

The anti-H3K56ac, anti-H3K27ac, and anti-H3K9ac antibodies were purchased from GeneTex (Irvine, CA 92606, USA). Anti-H3K14ac antibodies from Millipore (Billerica, MA 01821, USA), whereas anti-Chk1 antibodies, anti-pChk2 antibodies, anti-TLK1 antibodies, anti-Asf1a, and anti-Asf1b antibodies were purchased from Cell Signaling (Danvers, MA 01923, USA). Antibodies against β-actin and GAPDH were purchased from Santa Cruz Biotechnology (Dallas, TX 75220, USA). The anti-SNAP antibody was obtained from New England Biolabs (Ipswich, MA 01938, USA).

### Cellular Protein Fractionation, Immunoprecipitation, and Western Blotting Analysis

The cellular protein fractionation experiments were conducted as described by Anindya et al. (28), with modifications. Briefly, cells (~10^7^) were lysed with 1 ml (~5× cell volume) of cytoplasmic lysis buffer (10 mM Tris-HCl [pH 7.9], 0.34 M sucrose 3 mM CaCl_2_, 2 mM magnesium acetate, 0.1 mM EDTA, 1 mM DDT, 0.5% NP-40, and a protease inhibitor cocktail). Nuclei were pelleted by centrifugation at 3,500 *g* for 15 min and washed with cytoplasmic lysis buffer without NP-40 and then lysed in 1 ml of nuclear lysis buffer (20 mM HEPES [pH 7.9], 3 mM EDTA, 10% glycerol, 1.5 mM MgCl_2_, 150 mM KOAc, and protease inhibitors). The nucleoplasmic fractions were separated by centrifugation at 15,000 *g* for 30 min and the pellets were designated as chromatin fraction. For further processing, the chromatin fraction pellets were resuspended in 0.2 ml of nuclease incubation buffer (150 mM HEPES [pH 7.9], 1.5 mM MgCl_2_, 150 mM KOAc, and protease inhibitors) and incubated with 50 U Benzonase (25 U/μl) for 30 min at room temperature. The soluble chromatin fraction was collected by centrifugation at 20,000 *g* for 30 min, while the insoluble chromatin fraction was dissolved by boiling in SDS sample buffer. Soluble chromatin fractions may be kept frozen in storage buffer (10 mM Tris-HCl, 300 mM NaCl, 1 mM DTT, 0.1 mM EDTA, and 50% glycerol) at −20°C short term/or −80°C.

The immunoprecipitation was done at 4°C overnight in RIPA buffer (50 mM Tris-HCl [pH 8.0], 150 mM NaCl, 1% NP40, 0.5% deoxycholate, and protease inhibitors) using nuclease-releasable chromatin containing ∼200–500 μg protein, with proper amount of desired antibody and Protein A plus G beads. After 4°C incubation, the beads were washed 1 time with RIPA buffer and then 3 times with TBS buffer (50 mM Tris-HCl [pH 7.4] and 150 mM NaCl), and the bound proteins were eluted by boiling in SDS loading buffer.

The Western blotting analysis was done using SDS-PAGE, followed immuno-detection. To analyze histone, tagged histone variants, and their acetylation levels, the immunoprecipitation samples or chromatin fraction samples containing ~10–30 μg protein in SDS sample buffer were separated by 14% acrylamide gel, Western transferred to PVDF membrane, and immuno-detected with anti-SNAP, or acetylation site-specific histone antibodies and chemiluminescence.

### mRNA Sequencing

The RNA samples were extracted using a TRIzol reagent (Invitrogen, Thermo Fisher Scientific) from ~5 × 10^6^ cells according to the manufacturer’s instructions. The concentration of RNA was measured using a Qubit^®^ RNA Assay Kit and a Qubit^®^ 2.0 Fluorometer (Life Technologies, Thermo Fisher Scientific). The RNA integrity was assessed by the RNA Nano 6000 Assay Kit of the Bioanalyzer 2100 system (Agilent Technologies Santa Clara, CA 95051, USA).

Sequencing libraries were constructed using the NEB Next^®^Ultra^®^Directional RNA Library Prep Kit for Illumina^®^ (NEB) according to the manufacturer’s instructions by Biomarker Technologies Co. (Beijing, China). Briefly, mRNA was denatured by heating to 94°C for 15 min in 5× NEB Next First Strand Synthesis Reaction Buffer. Random primers, reverse transcriptase, and murine RNase inhibitor were then added, and first-strand cDNA was synthesized at 42°C for 30 min. Then, second-strand cDNA was synthesized using a synthesis enzyme mix for 60 min at 16°C and then for 30 min at 20°C. The resulting dsDNA fragments were purified using Agencourt AMPure XP Beads (Beckman Coulter, Beverly, CA, USA). The overhangs were digested to blunt ends with NEB Next End Prep Enzyme Mix and then adaptors linked to the USER Enzyme were ligated to the cDNA. The cDNA was then purified using AMPure XP Beads. Finally, the DNA fragments were amplified using Hot Start HiFi PCR Master Mix, and the products were re-purified using the AMPure XP system and library quality was analyzed using an Agilent Bioanalyzer 2100 and qRT-PCR. TruSeq PE Cluster Kitv3-cBot-HS was adopted to construct clusters of index-coded samples on an acBot Cluster Generation System (Illumina Inc., San Diego, CA, USA). The RNA library was then sequenced on an Illumina Hiseq platform and paired-end reads were generated.

The resultant raw reads were further cleaned by eliminating adapter sequences, low-quality reads, and poly-N sequences. The GC content and sequence duplication levels of the clean data were then calculated to confirm the quality of the data. For further analysis, Clean Reads of each sample were compared with reference annotation (ftp://ftp.ensembl.org/pub/release-95/fasta/homo_sapiens/dna/). Cufflinks package was used to calculate expression of genes depending on fragments per kilobase of exon per million reads (FPKM) values. DESeq R package (version 1.18.0) was used for statistical analysis of differential gene expression between samples. The *p*-values were set to <0.05 based on Benjamini and Hochberg’s method to reduce the false discovery rate. Genes with a log2 fold expression variation value > 1.5 were considered to be differentially expressed.

### Reverse Transcription and Quantitative Real-Time PCR

Reverse transcription was conducted with All-in-One™ First-Strand cDNA Synthesis kit (AORT-0060, GeneCopoeia, Shenzhen, China). To quantify the mRNA expression of HOXB6, DTX3L, SSTR2, MYC, and HOXC10, real-time PCR was performed on CFX96 Touch™ Real-Time PCR Detection System (Bio-Rad, Hercules, CA 94547, USA) using All-inOne™ mRNA Detection kit (AOPR-0200, GeneCopoeia) based on SYBR-Green. PCR primers for GAPDH (HQP006940), HOXB6 (HQP008992), DTX3L (HQP003638), SSTR2 (HQP017744), MYC (HQP117877), and HOXC10 (HQP009003) were all purchased from GeneCopoeia. In each qRT-PCR, GAPDH mRNAs were used as internal reference for normalization, respectively. The relative expression was calculated using the 2^−ΔΔCt^ method.

### Quantitative Analysis and Statistics

Quantitative analysis was done on digitalized Western blotting images by ImageJ software and the relative protein amounts were calculated based on gray density. The Student’s *t*-test was performed using Sigma Plot.

## Results

### UV Radiation Transient Suppresses H3.1K56ac and H3.3K56ac Levels and Their Restoration Following DNA Damage Repair

**Figure 1:**
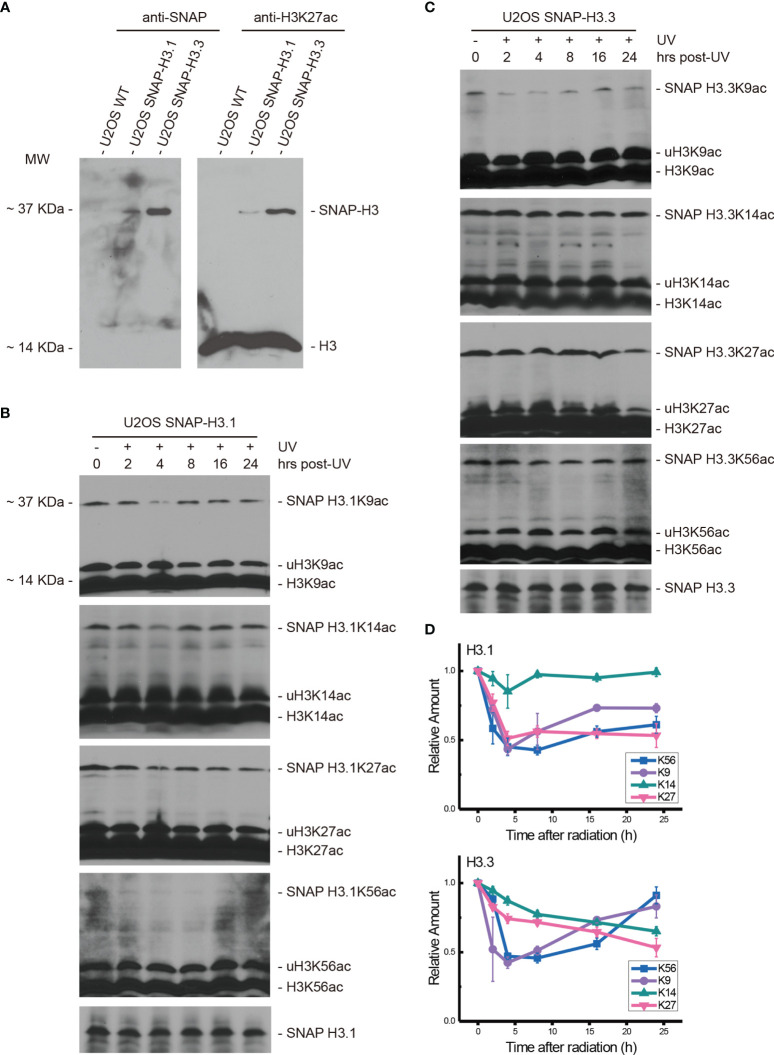
Both H3.1K56ac and H3.3K56ac were transiently suppressed by UV radiation and restored following DNA damage repair. **(A)** SNAP-tagged histone H3.1 and H3.3 were investigated in wild-type U2OS cells and H3.1-SNAP U2OS and H3.3-SNAP U2OS cells by an anti-SNAP antibody and anti-H3K27ac antibody. Bands at approximately 37 kDa indicate SNAP-tagged histone H3 or SNAP-tagged lysine 27 acetylated histone H3, respectively. **(B)** H3.1-SNAP U2OS cells were treated with 20 J/m^2^ UV radiation. The insoluble nuclear protein samples were collected at 0 h, 2 h, 4 h, 8 h, 16 h, and 24 h after radiation treatment. The acetylation levels of H3K9, H3K14, H3K27, and H3K56 were examined. SNAP-tagged histone H3 was used as an internal reference. **(C)** H3.3-SNAP U2OS cells were treated with 20 J/m^2^ UV radiation. The insoluble nuclear protein samples were collected at different time points after radiation treatment. The acetylation levels of H3K9, H3K14, H3K27, and H3K56 were investigated, and SNAP-tagged histone H3 was used as an internal reference. **(D)** Time-course alteration of acetylated K9, K14, K27, and K56 of H3.1 or H3.3 after 20 J/m^2^ UV radiation. The data are normalized immunoblotting results by ImageJ analysis from three separate experiments.

We previously demonstrated that the nucleotide excision repair factor CRL4^DDB2^ ubiquitin ligase preferentially regulates post-repair chromatin restoration of H3K56Ac (16). To distinguish between acetylated H3.1 and H3.3, two variants of histone H3 in such a post-repair chromatin restoration of H3K56Ac, we took advantage of the system, in which SNAP-tagged H3.1 and H3.3 were expressed in U2OS cells. This system was used to visualize H3.1 and H3.3 at UV damage repair sites (26). In such a study, the accumulation of new H3.1 at UV damage repair sites was demonstrated to be dependent on the H3.1 histone chaperone CAF1, while the accumulation of new H3.3 was dependent on H3.3 histone chaperone HIRA. In our study, H3.1-SNAP-tagged and H3.3-SNAP-tagged U2OS cells were cultured and verified by anti-SNAP and anti-H3K27ac antibodies. As shown in **Figure 1A**, the acetylated H3K27 band at approximately 14 kDa was detected in all three kinds of cells. SNAP-tagged H3.1 and H3.3 were detected as ~37-kDa bands only in H3.1- and H3.3-SNAP-tagged U2OS cells but not in SNAP-free U2OS cells. As expected, a similar pattern was found for SNAP-tagged H3K27ac. As for the difference in expression levels in SNAP-tagged H3.1 and H3.3, the band of SNAP-tagged H3.3 was more intense than that of SNAP-tagged H3.1. Similarly, the band of SNAP-tagged H3.3K27ac was also heavier than SNAP-tagged H3.1K27ac. Furthermore, the bands of histone H3 were also much heavier than the band of SNAP-tagged histone H3, presumably due to the low expression levels of SNAP-tagged H3.1 and H3.3 in U2OS cells.

After 20 J/m^2^ UV radiation, the time courses of H3.1K56ac and H3.3K56ac were investigated by Western blotting in H3.1- and H3.3-SNAP-tagged U2OS cells at 0, 2, 4, 8, 16, and 24 h after radiation. The acetylation levels of K27, K14, and K9 on histone H3.1 and H3.3 were measured at the same time points with the levels of SNAP-tagged H3.1 or H3.3 tested as the internal reference.

As shown in **Figures 1B, D**, the levels of H3.1K56ac, H3.1K27ac, and H3.1K9ac were decreased at the beginning after UV radiation, while the levels of H3.1K14ac were not significantly affected. The lowest acetylation level of H3.1K56 appeared at approximately 8 h after irradiation while the lowest H3.1K27ac and H3.1K9ac levels appeared at approximately 4 h after irradiation. After reaching the lowest point, the levels of H3.1K56ac and H3.1K9ac started increasing, which coincides with our previous study (16). The acetylation of H3.1K27 remained steady after reaching the lowest level until 24 h after radiation (**Figures 1B, D**). On the other hand, the acetylation levels of H3.3K56, H3.3K27, H3.3K14, and H3.3K9 were decreased by UV radiation, as shown in **Figures 1C, D**. The lowest H3.3K56ac and H3.3K9ac appeared 4–8 h after irradiation and then started to reverse, while the levels of H3.3K27ac and H3.3K14ac continued to decrease until 24 h after radiation.

### Singular ATM Inhibitor or ATR Inhibitor Treatment Did Not Influence the Patterns of H3.1K56ac and H3.3K56ac After UV Radiation

To determine the influence of ATM and ATR on the acetylation of H3.1K56 and H3.3K56 after UV radiation, the ATM inhibitor Ku55933 (10 μM) and the ATR inhibitor VE-821 (10 μM) were used, respectively. Both H3.1- and H3.3-SNAP expressing U2OS cells were treated with inhibitors individually and cellular fractionation samples were collected at 0, 2, 4, 8, 16, and 24 h after 20 J/m^2^ UV radiation. Histone acetylation was then examined in chromatin fractions.

**Figure 2:**
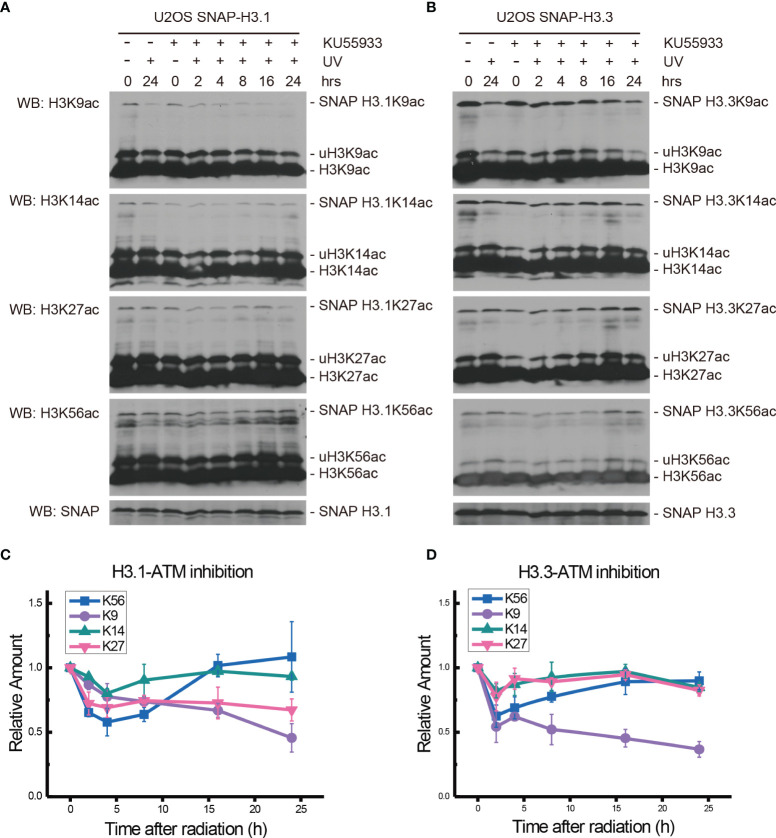
Modulation of acetylated K9, K14, K27, and K56 of histone H3.1 or H3.3 by the ATM inhibitor KU55933 and UV radiation. **(A)** H3.1-SNAP U2OS cells were pretreated with DMSO (-) or KU55933 (+) for 1 h and then irradiated with 20 J/m^2^ UV radiation. The insoluble nuclear protein samples were collected at 0 h, 2 h, 4 h, 8 h, 16 h, and 24 h after radiation. The acetylation levels of H3K9, H3K14, H3K27, and H3K56 were examined. SNAP-tagged histone H3 was used as an internal reference. **(B)** H3.3-SNAP U2OS cells were pretreated with DMSO or KU55933 and then irradiated with 20 J/m^2^ UV radiation. The insoluble nuclear protein samples were collected, and the acetylation levels of H3K9, H3K14, H3K27, and H3K56 were examined. SNAP-tagged histone H3 was used as an internal reference. **(C)** Time-course alteration of acetylated K9, K14, K27, and K56 of histone H3.1 after 20 J/m^2^ UV radiation and KU55933 treatment. The data are normalized immunoblotting results by ImageJ analysis from three separate experiments. **(D)** Time-course alteration of acetylated K9, K14, K27, and K56 of histone H3.3 after 20 J/m^2^ UV radiation and KU55933 treatment. The data are normalized immunoblotting results by ImageJ analysis from three separate experiments.

After Ku55933 treatment, the levels of H3.1K9ac decreased continually until 24 h after UV radiation (**Figures 2A, C**). At the same time, the acetylation of H3.1K14 showed a decrease after radiation and the lowest level appeared at approximately 4 h after radiation. Then, the H3.1K14ac levels were restored and remained steady until 24 h (**Figures 2A, C**). Combined with Ku55933 and UV radiation treatment, levels of H3.1K27ac and H3.1K56ac were also decreased, while the patterns of H3.1K27ac and H3.1K56ac were the same as those in radiation samples from treated cells, which are shown in **Figures 1B, D**. The H3.1K27ac level has bottomed out at approximately 4 h after radiation and then remained steady at this level for 24 h. The lowest H3.1K56ac also appeared at approximately 4 h after UV irradiation and then the acetylation levels started rising and returned to normal levels at approximately 16 h after radiation and continued to rise slowly for 24 h (**Figures 2A, C**).

As shown in **Figures 2B, D**, in the ATM inhibitor Ku55933-treated SNAP-tagged H3.3 U2OS cells, the levels of H3.3K9ac, H3.3K14ac, H3.3K27ac, and H3.3K56ac all decreased after UV radiation. However, H3.3K14ac and H3.3K27ac transiently decreased at 2 h after radiation and quickly recovered. The decrease in H3.3K9ac also began 2 h after radiation, while the acetylation levels showed a slight increase at 4 h and then continued to decrease until 24 h after UV radiation. The acetylation of H3.3K56 decreased 2 h after radiation and then continued to slowly rise until 24 h after radiation. This pattern was quite similar in simple irradiated cells without the Ku55933 treatment.

**Figure 3:**
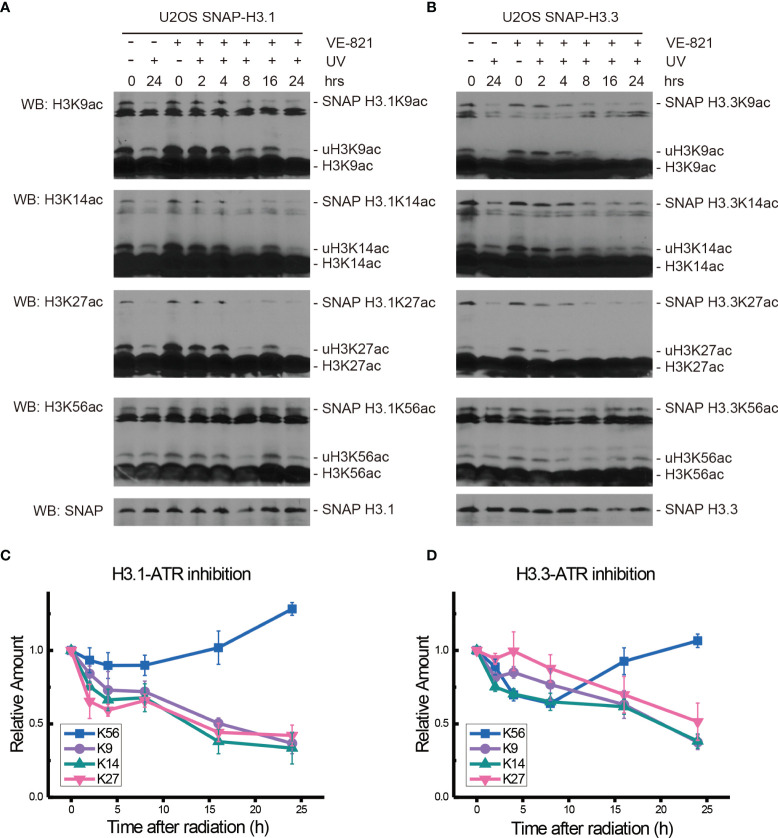
Modulation of acetylated K9, K14, K27, and K56 of histone H3.1 or H3.3 by the ATR inhibitor VE-821 and UV radiation. **(A)** H3.1-SNAP U2OS cells were pretreated with DMSO (-) or VE-821 (+) for 1 h and then irradiated with 20 J/m^2^ UV radiation. The insoluble nuclear protein samples were collected at 0 h, 2 h, 4 h, 8 h, 16 h, and 24 h after radiation. The acetylation levels of H3K9, H3K14, H3K27, and H3K56 were examined. SNAP-tagged histone H3 was used as an internal reference. **(B)** H3.3-SNAP U2OS cells were pretreated with DMSO or VE-821 and then irradiated with 20 J/m^2^ UV radiation. The insoluble nuclear protein samples were collected, and the acetylation levels of H3K9, H3K14, H3K27, and H3K56 were examined. SNAP-tagged histone H3 was used as an internal reference. **(C)** Time-course alteration of acetylated K9, K14, K27, and K56 of histone H3.1 after 20 J/m^2^ UV radiation and VE-821 treatment. The data are normalized immunoblotting results by ImageJ analysis from three separate experiments. **(D)** Time-course alteration of acetylated K9, K14, K27, and K56 of histone H3.3 after 20 J/m^2^ UV radiation and VE-821 treatment. The data are normalized immunoblotting results by ImageJ analysis from three separate experiments.

In the ATR inhibitor VE-821-treated H3.1-SNAP-tagged U2OS cells, the levels of H3.1K9ac, H3.1K14ac, H3.1K27ac, and H3.1K56ac all decreased during the first 4 h after UV radiation. The levels of H3.1K9ac, H3.1K14ac, and H3.1K27ac continued to decrease until 24 h after UV radiation, while H3.1K56ac started to rise from 8 h to 24 h after radiation (**Figures 3A, C**).

As shown in **Figures 3B, D**, the levels of H3.3K9ac, H3.3K14ac, H3.3K27ac, and H3.3K56ac in VE-821 treated H3.3-SNAP-tagged U2OS cells all decreased at 2 h after UV radiation. After that, the levels of H3.3K14ac continued to decrease until 24 h while the acetylation of H3.3K9 and H3.3K27 showed a slight increase and then continued to decrease. On the other hand, the decrease in H3.3K56ac continued for 8 h and then started to rise until 24 h after radiation.

### ATM and ATR Double Inhibition Suppressed the Restoration of H3.1K56ac and H3.3K56ac After UV Radiation

The ATM inhibitor Ku55933 and ATR inhibitor VE-821 were then used together to further probe the function of ATM and ATR in the acetylation of H3.1 and H3.3 in SNAP-tagged H3 U2OS cells. One hour after inhibitor pretreatment, cells were irradiated with 20 J/m^2^ UV and histone acetylation in U2OS cells was then examined in chromatin fractions.

**Figure 4:**
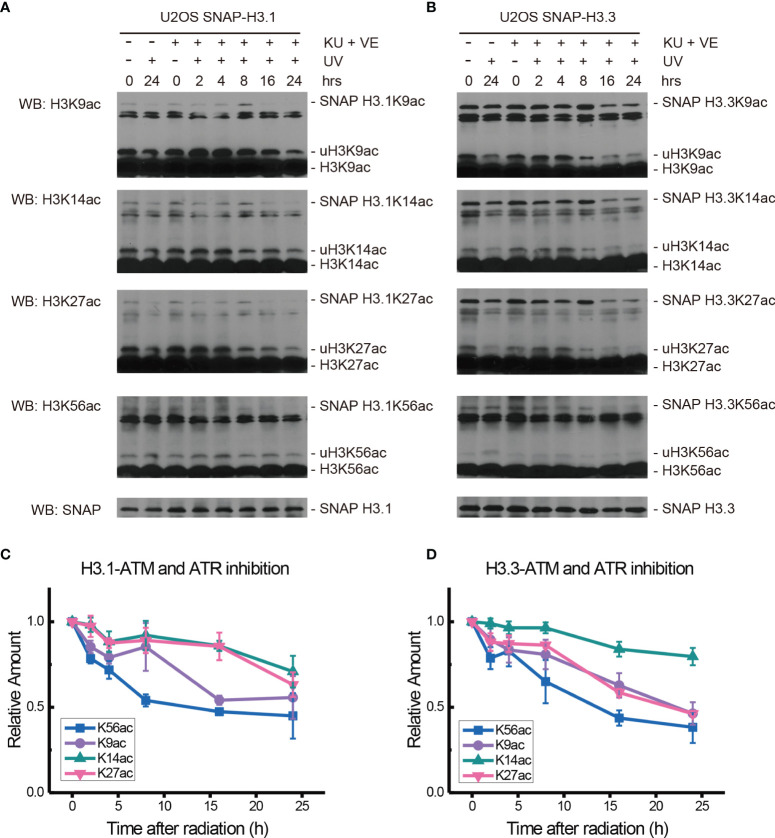
Modulation of acetylated K9, K14, K27, and K56 of histone H3.1 or H3.3 by an ATM inhibitor, ATR inhibitor, and UV radiation. **(A)** H3.1-SNAP U2OS cells were pretreated with DMSO (-) or KU55933 and VE-821 (+) for 1 h and then irradiated with 20 J/m^2^ UV radiation. The insoluble nuclear protein samples were collected at 0 h, 2 h, 4 h, 8 h, 16 h, and 24 h after radiation. The acetylation levels of H3K9, H3K14, H3K27, and H3K56 were examined. SNAP-tagged histone H3 was used as an internal reference. **(B)** H3.3-SNAP U2OS cells were pretreated with DMSO or two inhibitors and then irradiated with 20 J/m^2^ UV radiation. The insoluble nuclear protein samples were collected, and the acetylation levels of H3K9, H3K14, H3K27, and H3K56 were examined. SNAP-tagged histone H3 was used as an internal reference. **(C)** Time-course alteration of acetylated K9, K14, K27, and K56 of histone H3.1 after 20 J/m^2^ UV radiation and two inhibitor treatments. The data are normalized immunoblotting results by ImageJ analysis from three separate experiments. **(D)** Time-course alteration of acetylated K9, K14, K27, and K56 of histone H3.3 after 20 J/m^2^ UV radiation and two inhibitor treatments. The data are normalized immunoblotting results by ImageJ analysis from three separate experiments.

As shown in **Figures 4A, C**, after double inhibitor treatment, the acetylation of H3.1K9, H3.1K14, H3.1K27, and H3.1K56 under UV radiation decreased for 4 h after radiation. The levels of H3.1K9ac increased at approximately 8 h after radiation and quickly decreased at 16 h after radiation. The levels of H3.1K14ac and H3.1K27ac also showed a slight increase at approximately 8 h after radiation and then continued to slowly decrease for 24 h. In contrast, the levels of H3.1K56ac continued to decrease for 24 h after UV radiation, and the recovery of H3.1K56ac was absent. As shown in **Figures 4B, D**, the levels of H3.3K9ac, H3.3K14ac, H3.3K27ac, and H3.3K56ac all decreased 2 h after a double inhibitor treatment plus UV radiation. After that, the levels of H3.3K9ac, H3.3K14ac, and H3.3K27ac continued to decrease slowly until 24 h after radiation. However, H3.3K56ac showed a short increase at 4 h after UV irradiation and then continued to decrease slowly for 24 h. The recovery of H3.3K56ac under double inhibition was again absent.

### Chk1 Inhibition Blocked the Restoration of H3.1K56ac and H3.3K56ac Levels After UV Radiation

**Figure 5:**
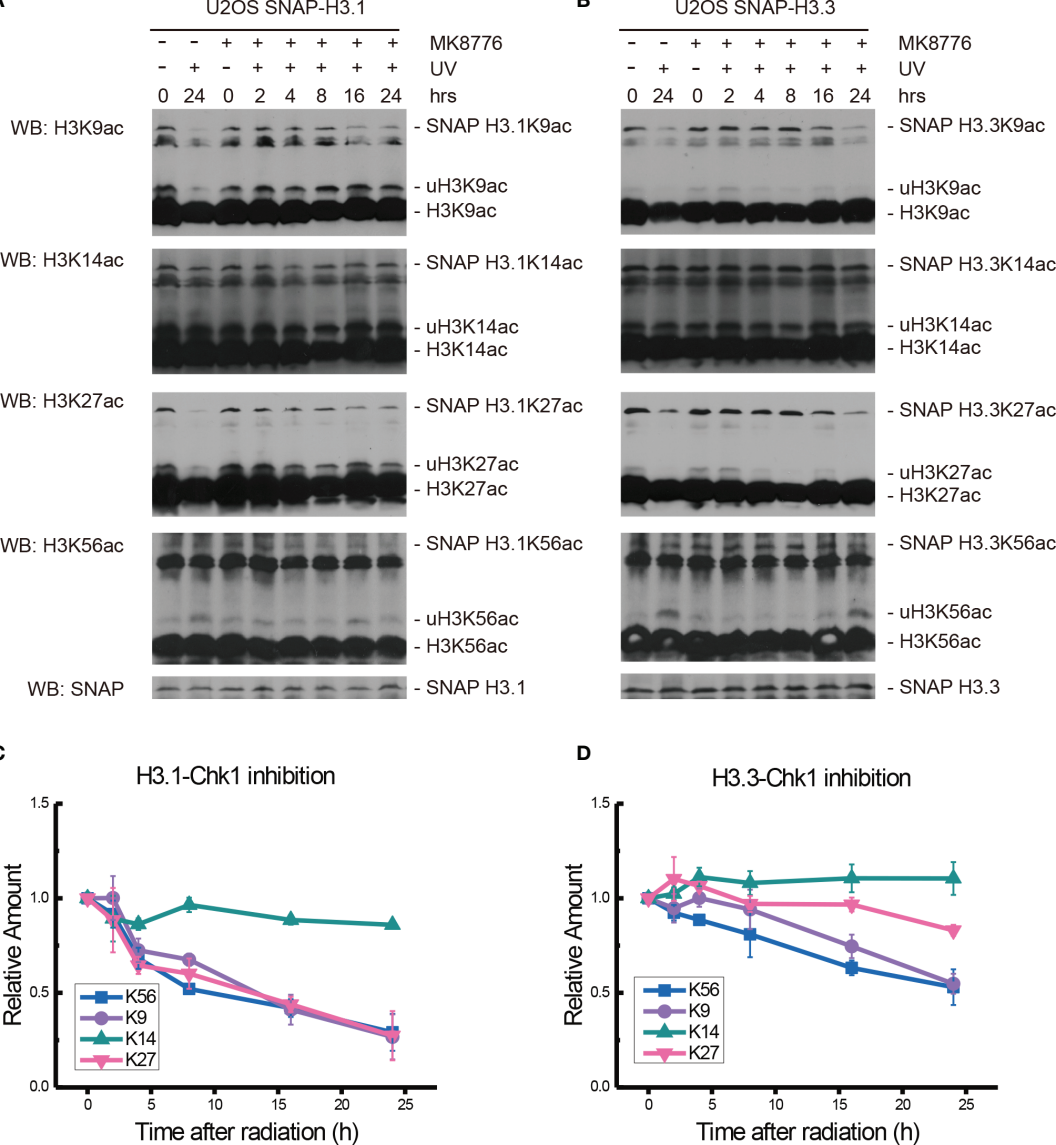
Modulation of acetylated K9, K14, K27, and K56 of histone H3.1 or H3.3 by the Chk1 inhibitor MK8776 and UV radiation. **(A)** H3.1-SNAP U2OS cells were pretreated with DMSO (-) or MK8776 (+) for 1 h and then irradiated with 20 J/m^2^ UV radiation. The insoluble nuclear protein samples were collected at 0 h, 2 h, 4 h, 8 h, 16 h, and 24 h after radiation. The acetylation levels of H3K9, H3K14, H3K27, and H3K56 were examined. SNAP-tagged histone H3 was used as an internal reference. **(B)** H3.3-SNAP U2OS cells were pretreated with DMSO or MK8776 and then irradiated with 20 J/m^2^ UV radiation. The insoluble nuclear protein samples were collected, and the acetylation levels of H3K9, H3K14, H3K27, and H3K56 were examined. SNAP-tagged histone H3 was used as an internal reference. **(C)** Time-course alteration of acetylated K9, K14, K27, and K56 of histone H3.1 after 20 J/m^2^ UV radiation and MK8776 treatment. The data are normalized immunoblotting results by ImageJ analysis from three separate experiments. **(D)** Time-course alteration of acetylated K9, K14, K27, and K56 of histone H3.3 after 20 J/m^2^ UV radiation and MK8776 treatment. The data are normalized immunoblotting results by ImageJ analysis from three separate experiments.

To explore the possible effectors downstream of ATM and ATR, the Chk1 inhibitor MK8776 at 0.5 μM was added 2 h before 20 J/m^2^ UV radiation, cellular fractionation samples were collected, and histone acetylation in chromatin fractions was again examined, as shown in **Figure 5**.

After MK8776 treatment and UV irradiation, the acetylation of H3.1K14, H3.1K27, and H3.1K56 all decreased remarkably, while the change in H3.1K9 was not obvious at 2 h after UV radiation (**Figures 5A, C**). The levels of H3.1K9, H3.1K27, and H3.1K56 sharply decreased at 4 h and then continued to decrease slowly until 24 h after radiation. The acetylation of H3.1K14 continued to decrease at 4 h and remained low until 24 h after UV radiation (**Figures 5A, C**). As shown in **Figures 5B, D**, the levels of H3.3K9ac and H3.3K56ac decreased while the levels of H3.3K14ac and H3.3K27ac increased at 1 h after UV radiation. Then, the levels of H3.3K27ac and H3.3K56ac continued to decrease for 24 h. The levels of H3.3K9ac experienced a short increase at 4 h and then continued to decrease slowly for 24 h. The levels of H3.3K14ac increased slightly at 4 h and then remained steady until 24 h after UV radiation (**Figures 5B, D**).

**Figure 6:**
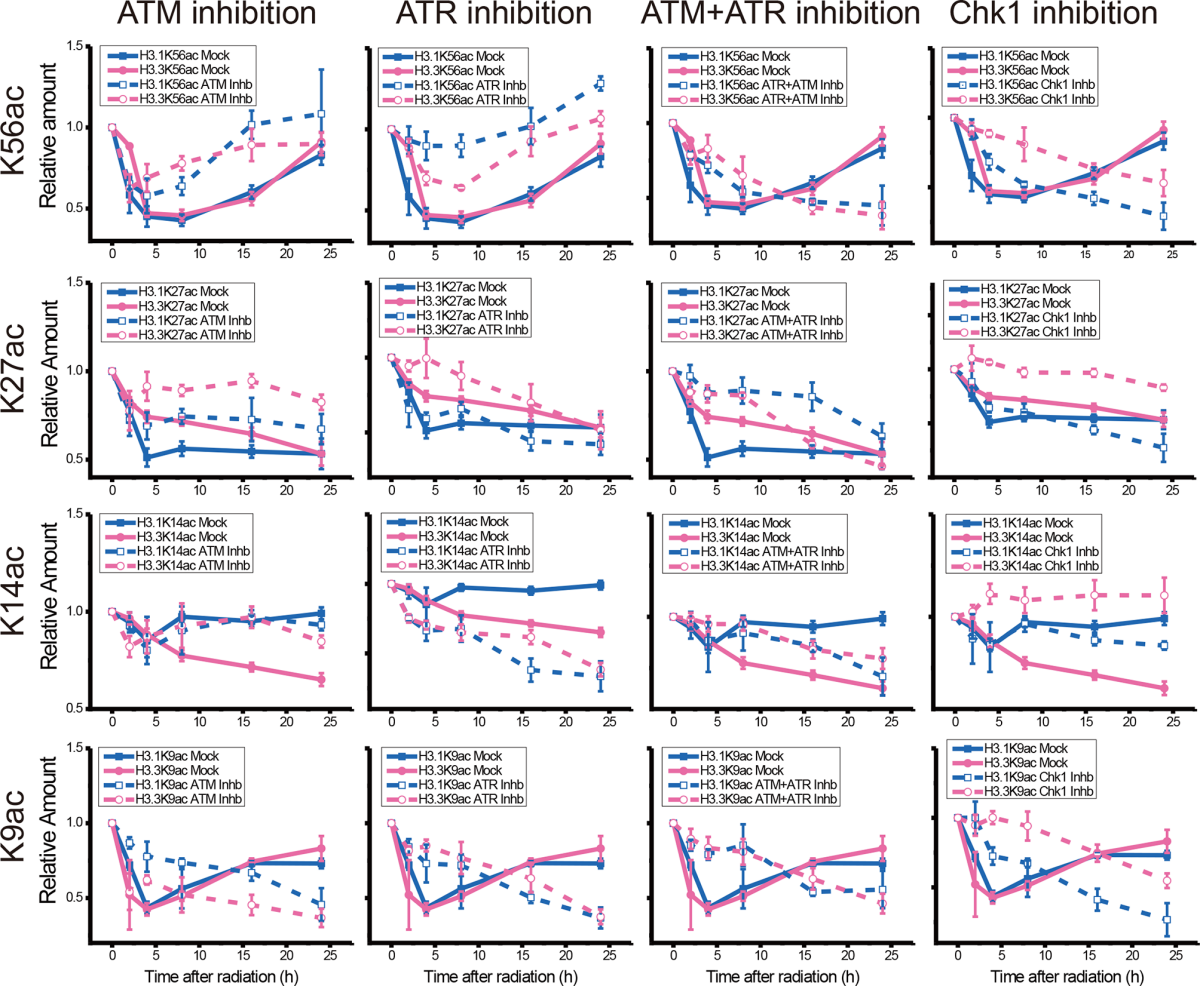
Comparison of the acetylation levels of K9, K14, K27, and K56 of histone H3.1 and H3.3 in irradiated U2OS cells. For each acetylation site, the relative amounts of H3.1 and H3.3 acetylation before and after chemical treatment were compared separately.

While the individual ATM inhibition by Ku55933 or ATR inhibition by VE-821 did not suppress the restoration of histone H3K56 acetylation levels, both individual inhibitions indeed suppressed the decrease in H3.1K9ac, H3.3K9ac, H3.1K27ac, H3.3K27ac, H3.1K56ac, and H3.3K56ac after UV radiation, especially in the first 8 h after radiation (**Figure 6**). In contrast, Chk1 inhibition by MK8776 resulted in a decrease in acetylation at all tested sites. The acetylation levels were higher than those in cells without inhibitor treatment and the influence of MK8776 on H3.3 was more serious than that on H3.1. However, this phenomenon was not observed under the Ku55933 plus VE-821 treatment (**Figure 6**).

### Chk1 Inhibition Results in Cellular Transcription Suppression

We further examined the influence of Chk1 inhibition on patterns in cellular gene expression during UV DNA damage repair. The U2OS cells were pretreated with vehicle DMSO or the Chk1 inhibitor Ly2606368 at 10 nM 1 h before 20 J/m^2^ UV radiation. At 2, 8, and 24 h after UV radiation, RNA samples were collected for RNA sequencing. Nineteen libraries were generated from our experimental groups (Sequencing ID: BMK190830-U285-0102), and summaries of the RNA sequencing analyses are shown in **Tables S1**, **S2**. More than 38,339,762 reads (from 38,339,762 to 50064758) were sequenced from each library, and more than 96.44% (from 96.44% to 97.38%) clean reads were unique reads, of which more than 89.79% (from 89.79% to 94.59%) reads were paired reads that mapped to the human genome.

**Figure 7:**
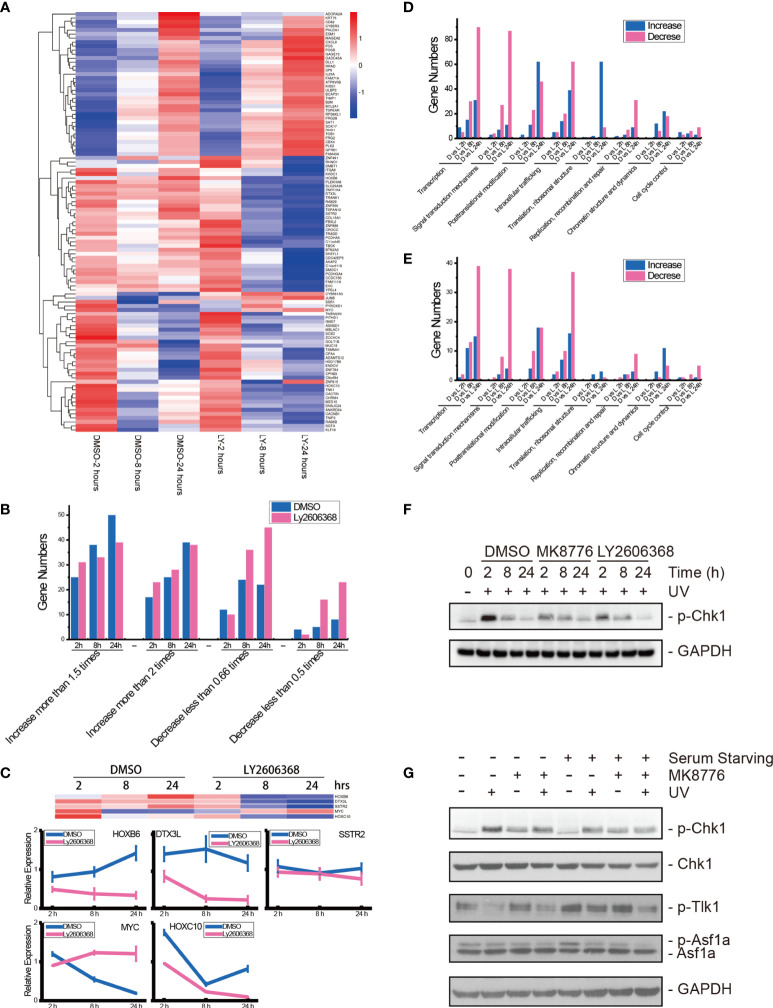
mRNA sequencing of Chk1-inhibited samples after UV radiation and verification of Chk1 inhibition. **(A)** Heatmap visualization of 105 genes that were expressed in all libraries. Data are expressed as the mean FPKM, standardized, and visualized by the DESeq R package (version 1.18.0). Columns: samples; rows: genes; red: relatively high expression; blue: relatively low expression. DMSO: DMSO mock group; LY: Ly2606368 treated group; 2 hours: 2 h after UV radiation; 8 hours: 8 h after UV radiation; 24 hours: 24 h after UV radiation. **(B)** Calculations of differentially expressed genes in the 105 genes. **(C)** qRT-PCR results of HOXB6, DTX3L, SSTR2, MYC, and HOXC1 mRNA expression levels after DMSO or Ly2606368 treatment and UV radiation. **(D)** Numbers of changed genes after chemical treatment and UV radiation. At 2 h, 8 h, and 24 h after UV radiation and chemical treatment, the normalized gene expression of Ly2606368-treated U2OS cells and DMSO-mock U2OS cells was compared, and the numbers of differentially expressed genes classified by functional annotation are shown. **(E)** Numbers of genes that changed more than 2-fold after chemical treatment and UV radiation. The numbers of genes that increased more than 2-fold or decreased more than 0.5-fold were calculated. **(F)** Western blot detection of phosphorylated Chk1 after DMSO, MK8776, or Ly2606368 treatment after UV radiation. The data are normalized immunoblotting results by ImageJ analysis from three separate experiments. **(G)** Western blot detection of phosphorylated Chk1, Tlk-1, and Asf1a after DMSO or MK8776 treatment under serum starvation conditions and UV radiation. For the serum starvation experiment, U2OS cells were seeded and grown to 70% confluence, and then the medium was changed to serum-free DMEM for 24 h.

There were 105 genes that were expressed in all 19 libraries. The relative expression levels of these 105 genes are listed in a heatmap (**Figure 7A** and **Table S3**). Compared with the controls, approximately two-thirds of the genes were upregulated at 2 h following UV radiation in both the DMSO-mock group and the Ly2606368-treated group. At 8 and 24 h, more genes were downregulated in the Ly2606368-treated group. However, expression resumed for more genes in the mock, and small sections of them were even upregulated at 8 h and 24 h. The numbers of upregulated or downregulated genes among these 105 genes were calculated (**Figure 7B**). Several genes with expression that increased at least two times showed little difference between the DMSO-mock group and the Ly2606368-treated group. When the threshold was extended to 1.5 times, the number of upregulated genes in the DMSO-mock group was slightly higher than that in the Ly2606368-treated group. On the other hand, there were more downregulated genes at the 8- and 24-h time points in the Ly2606368-treated group than in the DMSO-mock group under either 0.66- or 0.5-times threshold. The sequencing results were confirmed by qRT real-time PCR, and the trends of DNA repair- or translation-related genes such as HOXB6, DTX3L, SSTR2, MYC and HOXC10 were coincident with the mRNA sequencing results (**Figure 7C**).

We further compared the expression of genes in the Ly2606368-treated group with that in the DMSO-mock group at the same time point after UV radiation, and the number of differentially expressed genes (DEGs) was calculated and categorized by eggNOG class annotation (**Figures 7D, E**and **Table S4**). Analysis of the functional gene distribution showed that transcription-, signal transduction mechanism-, posttranslational modification-, and intracellular trafficking-related genes were the most common DEGs. The transcription-related DEGs were the most abundant, and there were 90 decreased and 30 increased genes in the Ly2606368-treated group as compared with the DMSO-mock group at 24 h, 30 decreased and 15 increased genes at 8 h, and 5 decreased and 9 increased genes at 2 h. The number of decreased genes at 24 h was the highest (**Figure 7D**). In addition, a significant change (log2 more than 1 or less than −1) was also detected in transcription-related DEGs, and among these DEGs, there were 39 decreased and 15 increased genes (**Figure 7E**). The second most abundant DEGs were signal transduction mechanism-related genes, and among them, the number of decreased genes was higher than increased genes in the Ly2606368-treated group compared with the DMSO mock group at all time points (**Figures 7D, E**).

The changing status of Chk1 activation after inhibitor treatment and the influence of the Chk1 inhibitor on Tlk1 and Asf1 were verified by Western blotting. The levels of phosphorylated Chk1 (p-Chk1) were significantly upregulated after UV radiation and declined gradually until 24 h after UV radiation and the levels of p-Chk1 were remarkably suppressed by two special Chk1 inhibitors, MK8776 and Ly2606368. In addition, the levels of Chk1 remained stable under the same treatments (**Figure 7F**). Additionally, when the activation of Chk1 was induced by UV radiation, the levels of p-Tlk1 were suppressed, as were the levels of p-Tlk1 and p-Asf1a. As expected, the inhibition of p-Chk1 by MK8776 resulted in the upregulation of p-Tlk1 and p-Asf1a (**Figure 7G**).

## Discussion

Since the discovery of the unique H3K56 acetylation pathway by genetic screening in budding yeast (12), the acetylation of H3K56 has become an increasingly important phenomenon for understanding the mechanisms of chromatin dynamics in various cellular processes, such as transcription, DNA replication, and DNA damage response. In this study, we investigated the regulation of acetylation restoration at the 56 lysine residues of both histone H3.1 and H3.3 by ATM, ATR, and Chk1 after UV radiation. According to our results, neither ATM nor ATR inhibition is capable of fully suppressing the restoration of either H3.1K56ac or H3.3K56ac after UV radiation. However, the Chk1 inhibition is abundant to suppress the restoration. Furthermore, total genome transcription was suppressed, and the expression of transcription-related genes was decreased by Chk1 inhibition. Given that ATM, ATR, and Chk1 are not directly involved in nucleotide excision repair of UV-induced DNA photolesions, our data suggest the key function of Chk1 in histone PTMs, chromatin dynamics and transcription regulation and recovery during DNA damage repair.

A large body of studies has revealed that histone acetylation, in addition to its role in transcriptional regulation, belongs to a broad repertoire of histone modifications involved in the DDR (14, 16, 29, 30). We and others have reported that H3K9ac and H3K56ac are highly responsive to DNA damage and repair (14, 16). Both H3K9ac and H3K56ac are rapidly and reversibly reduced after DNA damage, including UV radiation, ionizing radiation, and phleomycin. Furthermore, both histone H3.3 and H3.1 variants have been observed to be recruited to sites of DSB and UV damage (26, 31, 32). These findings revealed the participation of K9ac and K56ac of both H3.1 and H3.3 in nucleosome disassembly and reassembly during UV damage repair. As expected, we detected a rapid decrease and gradual restoration of H3.1K9ac, H3.3K9ac, H3.1K56ac, and H3.3K56ac after UV radiation (**Figure 1**). Our data confirmed that both H3.1-associated DNA synthesis-dependent and H3.3-associated DNA synthesis-independent but transcription-dependent chromatin dynamics occur during UV damage repair. Our data further revealed that both H3.1 and H3.3 are regulated by acetylation, and the latter are further controlled by Chk1 and by ATM and ATR kinases, upstream of Chk1, in a redundant manner.

It has been reported that DNA damage triggers a decrease in cellular H3K27ac levels (33) or induces an enrichment of H3K27ac at specific promoter regions (34). Accordingly, our data showed that the levels of both H3.1K27ac and H3.3K27ac decreased after UV radiation. H3K14ac is a non-DNA damage-responsive histone modification (14). Our results (**Figure 1**) showed that the levels of H3.1K14ac were steady after radiation while the levels of H3.3K14ac slightly decreased. However, it remains unclear whether such a subtle change is related to transcription-related chromatin dynamics. Taken together, our data verified the decrease and restoration of H3K56ac and H3K9ac after UV radiation. Our SNAP-based H3.1 and H3.3 PTMs detection system faithfully uncovered the changes in histone acetylation in these H3 variants and the possible regulation of acetylation by checkpoint kinases.

It is generally recognized that H3K56ac incorporation into repaired chromatin signals the completion of DNA repair as well as termination of checkpoint signaling activation. To relate H3.1 and H3.3 acetylation to these events, we examined the effect of kinase inhibition on H3 acetylation using the ATM inhibitor KU55933, ATR inhibitor VE-821, ATM and ATR inhibitor AZD6738, Chk1 inhibitor UCN-01, MK8776, Ly2606368, and Chk1 and Chk2 inhibitor AZD7762. In our experiments (**Figures 2**–**5** and summary **Figure 6**), the restoration of both H3.1K56ac and H3.3K56ac was not hindered by single ATM or ATR inhibition, while the ATM–ATR dual inhibition and Chk1 inhibition successfully suppressed the H3K56Ac restoration.

Chk1 is conventionally phosphorylated and activated by ATR, which is recruited by RPA-coated single-stranded DNA, and Chk1 acts downstream. ATM, however, is phosphorylated and activated by single DNA breaks, and Chk2 acts downstream of ATM upon single- and double-DNA breaks. However, Cep164 is phosphorylated by ATR/ATM both *in vivo* and *in vitro* which can induce phosphorylation of Chk1 upon replication stress and radiation (35). Our results suggest that Chk1 is a converging point through which ATM and ATR redundantly regulate the restoration of H3K56ac.

DNA damage is known to induce the rapid transcriptional repression and activation of a variety of genes related to cell cycle arrest, DNA damage repair, senescence, and apoptosis; Chk1 was reported to influence histone posttranslational modifications. For instance, Chk1 is responsible for the phosphorylation of H3T11 as the kinase that suppresses gene transcription following DNA damage *in vitro* (36). In our study, when the UV radiation-induced activation of Chk1 was suppressed by Ly2606368, the downregulation of gene expression was slow but continuous, especially at 24 h after treatment (**Figure 7**). Importantly, the largest group of downregulated genes were transcription-related genes (**Figures 7D, E**). These data revealed that Chk1 inhibition aggravated the inhibition of transcription related to cell cycle recovery. Moreover, UV radiation induced a rapid decrease in *c-myc* expression and then increased until 24 h after UV radiation, while Ly2606368 plus UV treatment strongly induced a decrease in *c-myc* expression. The data coincided with an earlier report that revealed the function of Ly2606368 in the suppression of both Chk1 and *c-myc* activity (37).

Taken together, the results of the present study identified Chk1 as an important histone H3K56 acetylation regulator during the DDR, by regulating the restoration of both H3.1K56ac and H3.3K56ac. More in-depth future studies to gain informative insights into how Chk1, Tlk1, and Asf1a interact with each other and function in histone PTMs would further clarify the mechanistic nature of these complex interactions in DNA damage processing.

## Data Availability Statement

The datasets presented in this study can be found in online repositories. The names of the repository/repositories and accession number(s) can be found in the article/**Supplementary Material**.

## Author Contributions

ND and QZ participated in the design of this study. ND, ZS, FY, and PQ conducted the experiments and analyzed and interpreted the data in this study. PL and DL performed statistical analysis. JW and QZ collected the background information. ND drafted the manuscript. ND, JW, and QZ provided funding support. All authors contributed to the article and approved the submitted version.

## Funding

This research was funded by the National Institute of Health of the USA (no. ES012991, QZ), the National Natural Science Foundation of China (no. U1932208, Kai Yang), the Science and Technology Research Project of Gansu Province (no. 17JR5RA307 and 145RTSA012, JW), and the Science and Technology Research Project of Gansu Province (no. 21JR7RA108, ND).

## Conflict of Interest

The authors declare that the research was conducted in the absence of any commercial or financial relationships that could be construed as a potential conflict of interest.

## Publisher’s Note

All claims expressed in this article are solely those of the authors and do not necessarily represent those of their affiliated organizations, or those of the publisher, the editors and the reviewers. Any product that may be evaluated in this article, or claim that may be made by its manufacturer, is not guaranteed or endorsed by the publisher.

## Supplementary Material

Supplementary Table 1 Sequencing summary of all samples.

Supplementary Table 2 Normalized reads of detected genes in each sample.

Supplementary Table 3 Fold changes in genes detected in all samples.

Supplementary Table 4 Relative expression of genes at 2 h, 8 h, and 24 h after radiation.
